# Effectiveness of the SBAR-Based training program in self-efficacy and clinical decision-making of undergraduate anesthesiology nursing students: a quasi-experimental study

**DOI:** 10.1186/s12912-023-01290-0

**Published:** 2023-04-27

**Authors:** Mehran Farzaneh, Vahid Saidkhani, Kambiz Ahmadi Angali, Masoumeh Albooghobeish

**Affiliations:** 1 Department of Anesthesiology, School of Allied Medical Sciences, Ahvaz Jundishapur University of Medical Sciences, Ahvaz, Iran; 2 Department of Nursing, School of Nursing and Midwifery, Ahvaz Jundishapur University of Medical Sciences, Ahvaz, Iran; 3 Biostatistics and Epidemiology Department, Health School, Ahvaz Jundishapur University of Medical Sciences, Ahvaz, Iran; 4 Department of Anesthesiology, School of Allied Medical Sciences, Ahvaz Jundishapur University of Medical Sciences, Ahvaz, Iran

**Keywords:** Anesthesiology nursing, Clinical decision-making, Clinical self-efficacy, Training program, SBAR

## Abstract

**Background:**

Structured Situation, Background, Assessment, and Recommendation (SBAR) training technique have been widely utilized in clinical and educational settings. Therefore, the current study investigated the effectiveness of an SBAR-based educational program in students’ self-efficacy and clinical decision-making skills.

**Methods:**

This quasi-experimental study was conducted using a pretest and posttest design and a control group at Ahvaz Jundishapur University of Medical Sciences, Ahvaz, Iran. A total of 70 three- and fourth-year students were recruited for the study using the census method. The students were randomly assigned to the intervention and control groups. The intervention group participated in an SBAR-based educational course consisting of eight sessions held in 4 weeks. Differences in the levels of self-efficacy and clinical decision-making skills before and after participation in the SBAR course were assessed and compared. Data were analyzed using descriptive tests, the Mann-Whiney U test, paired and independent t-tests, and the Wilcoxon test.

**Results:**

The intervention group demonstrated significantly higher levels of self-efficacy with a mean score of 140.66 ± 22.43 (P < 0.001) and clinical decision-making with a mean score of 75.31 ± 7.72 (P < 0.001); while in the control group, the mean score of self-efficacy and clinical decision-making skills was 85.34 ± 18.15 and 65.51 ± 4.49, respectively. Moreover, the Mann-Whitney U test showed that the levels of students’ clinical decision-making skills were promoted to the next level after the intervention (P < 0.001); it means the distribution of the level of intuitive-interpretive skill was upgraded from 0 to 22.9%.

**Conclusion:**

The SBAR-based training programs can promote the self-efficacy and clinical decision-making skills of anesthesiology nursing students. Considering the weakness of the anesthesiology nursing curriculum at the undergraduate level in Iran, it can be expected that the SBAR-based training course should be included as an educational intervention in the curriculum of anesthesiology nursing students.

## Introduction

Anesthesiology nurses are members of the operation theater nursing team who are in direct contact with patients from admission to discharge. Anesthesiology nurses deliver a wide range of clinical services, including preoperative assessments, intraoperative care, and postoperative care [1]. Anesthesiology nurses spend all their professional time in different clinical settings, and patients with different needs benefit from the experiences they have obtained during their training. Therefore, considering the aforementioned issue, the students need to be trained and evaluated in various clinical settings (including operation theater, post-anesthesia care unit (PACU), and intensive care unit (ICU)) [2]. In such environments, clinical decision-making skills and self-efficacy play a peculiar role in the provision of quality care [3].

Clinical decision-making is a major component of the nursing profession, including data analysis, decision-making, and application of these skills in a clinical context [4]. The prompt decision-making of nurses leads to shortening the treatment duration, promotion of care, and reduction of treatment costs. On the other hand, failure of timely and appropriate decision-making leads to delay in treatment and waste of resources and affects the quality of care [5]. Moreover, in any organizational context, nurses are members of a clinical team and cannot function independently. Teamwork needs ongoing decision-making, and these decisions can influence teamwork and determine the quality of care [6].

Different individual- and group-related factors can affect the decision-making potential of nurses, among which are self-belief and self-efficacy [7]. Self-efficacy plays a particular role in medical education and is considered to have an effective role in the integration of knowledge and attitude of nurses considering their potential in communication skills, data presentation, support, and self-management skills [8]. Several studies have demonstrated that self-efficacy plays an important role in the determination of the outcomes of training, particularly in delivering clinical care in undergraduate education [9, 10]. Therefore, nursing colleges endeavor to achieve their educational goals through training, guidance, and leadership of their tutors [11].

According to the Institute for Health Improvement (2020), Situation, Background, Assessment, and Recommendation (SBAR) is a simple mechanism for the establishment of effective dialogue, development of teamwork, and promotion of patient safety culture. Moreover, the application of the SBAR method plays a great role in the promotion of basic capacities, such as communication skills, clinical safety considerations, critical thinking, and problem-solving skills [12–14]. The successful implementation of the SBAR technique can ensure the satisfaction of both patients and the staff in high-risk environments, such as ICU, emergency departments, and operation theaters [15]. Moreover, using the SBAR technique is important to ensure patient safety, establish confidence, and promote collaboration with other medical staff in a real clinical context; therefore, its effective application in the nursing students’ curriculum is necessary [16].

While technical skills are thought to be the foundation of medical sciences education, nontechnical skills such as clinical and self-efficacy skills are also emerging phenomena in nursing education that receive less attention [17]. In addition, a bachelor’s degree in anesthesiology nursing is a novel major in medical education, and despite the increase in courses, particularly practical ones, the infrastructure needed for the implementation of the main educational goals and identification of shortcomings and deficiencies have not yet been realized. Additionally, a limited number of studies have been carried out in this field concerning anesthesiology nursing students.

Nonetheless, SBAR training has been effective in the promotion of different educational outcomes, such as the enhancement of nursing students’ competence and communicational skills [18], clinical competence [19], job satisfaction [20], and attitude of inter-professional collaboration [21].

The student’s ability to carry out practical therapeutic proficiencies similar to those used in real workplaces is greatly aided by profound simulation-based experiences combined with practical repetitions in non-stressful settings. This increases student’s confidence in their ability to make decisions and increases their self-efficacy in the field of anesthesia [22]. There is little research on clinical assessment in anesthesia nursing programs. Non-technical abilities may be taught and are not just gained by working for years in an intensive care unit, according to a research of first-year student registered nurse anesthetists (SRNAs) [23]. Students in anesthesiology nursing programs experience extreme stress, which can result in poor self-efficacy, or the belief that success is improbable. Enhancing self-efficacy consistently leads to greater academic performance, and its use in nurse anesthesia could be as promising [24].

The anesthesia nursing team members among the numerous professional teams in operating rooms need self-efficacy and clinical decision-making abilities to appropriately accomplish the expected therapeutic results. Self-efficacy and clinical decision-making capabilities, therefore, appear to be requirements for anesthetic services, in addition to technical capabilities. The educational curriculum for undergraduate anesthesiology nursing students does not, for the most part, include these factors. Thus, the current study was designed to assess the effectiveness of an SBAR-based training course in self-efficacy and clinical decision-making skills of anesthesiology nursing students.

## Methods

### Study Design

The current quasi-experimental study was conducted on undergraduate anesthesiology nursing students using a pretest and posttest design and a control group. In this study, an SBAR-based model was utilized as a framework for teaching self-efficacy and clinical decision-making skills to the students in an academic environment.

The SBAR training course was designed as an eight-session course consisting of 2-hour sessions (two sessions per week). This training course was held for the students in the intervention group as lectures, group discussions, question and answer sessions, scenario-writing assignments, and role-playing and debriefing. In the debriefing, the students described their experiences and any positive or negative aspects they noticed while role-playing various scenarios. To get accurate and appropriate feedback, the researcher and the learner examined and reviewed these thoughts and experiences. Therefore, the problematic points were identified and noted during debriefing sessions, while the positive parts were also highlighted and reinforced. In order to organize educational sessions for instructing the courses, four professional lecturers and faculty members from the university who are sufficiently knowledgeable in the specialized subjects offered to students were chosen. To provide supervision and ensure proper implementation of the program for the intervention group, the researcher attended the educational sessions and used a self-made checklist designed based on the SBAR technique in order to check the correct process of the SBAR training program. The content of each session was prepared based on the anesthesiology nursing curriculum accordingly, as shown in Table 1.

**Table 1:** Components of SBAR Training Program

Session	Content	Method
**First** (Situation)	• Completion of pretest questionnaires • Introduction of the course • Definition and the need for SBAR • SBAR components • Implementation methods and their advantages in clinical situations • Training (e.g., the importance of patient presentation, patient identification, and assessment of the current challenging situation and serious and urgent patient problems)	• Direction: Training objectives, using situation as one of the four elements of SBAR • Lectures • Group discussions and presentations • Questions and answers
**Second** (Background)	• Composition of SBAR • Training: 1) Comprehension of nursing highlights: the importance of an accurate diagnosis, patient history, awareness of allergies and sensitivities, importance of vital signs and laboratory reports, and summary of the completed tasks and their times 2) Complete understanding of challenging situations	• Using background as one of the four elements of SBAR • Lectures • Group discussions and presentations • Questions and answers
**Third** (Assessment)	• Composition of SBAR • Training: 1) Importance of clinical assessment by nurse anesthetists 2) Evaluation of the patient’s current condition 3) Analysis and a systematic classification of the information obtained from the patient’s history	• Using assessment as one of the four elements of SBAR • Lectures • Group discussions and presentations • Questions and answers
**Fourth** (Recommendation)	• Composition of SBAR • Planning outcomes and expected interventions for problem-solving in nursing • Planning for possible questions of the subjects • Making proposals for solving nurses’ problems • Training: 1) Tasks before anesthesia: Evaluation of the informed consent form, patient stress and anxiety management, examination of airways for identification of patients with difficult airways, preparation of the appropriate anesthesia instruments with regard to patients’ situations and the surgery (including the instruments used for airways management, anesthesia induction, monitoring, and positioning), securing intravenous lines, and assisting anesthesiologists in anesthesia induction and analgesia 2) Tasks during anesthesia: Checking vital signs, intravenous fluid administration, calculation of maximum allowable blood loss according to the aggressiveness of the operation, estimation of bleeding volume, and monitoring vital signs while bleeding (e.g., blood pressure, heart rate, and urine output) 3) Tasks after anesthesia: Assisting in awakening patients from anesthesia at the end of the surgery, safe transferring of patients to the PACU, communicating notable circumstances during the surgery to the PACU personnel, and delivering proper care in the recovery unit	• Using recommendation as one of the four elements of SBAR • Group discussions • Questions and answers
**Fifth** (Recommendation)		
**Sixth** (Role-playing)	• Review of all SBAR stages • Practice: (scenarios, such as presentation of clinical circumstances before, during, and after anesthesia, with the main objective of students’ self-efficacy and clinical decision-making, were developed.) 1) Dividing the students into 3-person groups 2) Trying to make correct decisions in the mentioned situations according to the scenarios using the SBAR model 3) Rehearsing based on the scenarios 4) practicing to improve scenarios through role-playing	• Writing scenarios and presenting information regarding role-playing • Role-playing (using the four elements of SBAR)
**Seventh** (Role-playing)		
**Eighth** (Debriefing and troubleshooting)	• Assessing the levels of the knowledge and receiving feedback • Highlighting shortcomings of the students in clinical decision-making and adherence to the SBAR technique • Focusing on clinical deduction and improvement of the judgment skills of the students • Discussing identified challenges before, during, and after anesthesia stages and rising to the challenges • Completing posttest questionnaires	• Group discussions and presentations • Debriefing • Reflection

### Sample and setting

The research sample consisted of 70 undergraduate anesthesiology nursing students of Ahvaz Jundishapur University of Medical Sciences who were recruited through the census method among the students in their third and fourth years. The allocation of the students was carried out on an equal and randomized basis. Using block randomization, students were divided into two blocks. Then, using random numbers generated by Excel, the students of each block were divided into two control and intervention groups.

According to the type of variables studied, we should have selected students experienced in the operating room environment and with sufficient knowledge of clinical settings. Therefore, only the third- and fourth-year students were included as they had passed at least one practical training course and were familiar with the clinical environment. Moreover, their evaluation in terms of clinical self-efficacy and clinical decision-making skills was possible. The inclusion criteria were willingness to participate in the course, understanding the goals and different processes of the research, and third- and fourth-year anesthesiology nursing students. Reluctance and departure from the study at any moment throughout the training session constituted exclusion criteria.

### Data collection

A questionnaire with three sections was used for data collection. The first section contained demographic data, including age, gender, academic year, academic performance, satisfaction with anesthesiology nursing major, and satisfaction with the practical training courses.

The second section consisted of Lauri & Salantera’s clinical decision-making scale (CDM) developed through a comprehensive literature search and several qualitative studies [25]. This tool contains 24 items based on a 5-point Likert scale (from 5 = always to 1 = never) to assess the clinical decision-making skills of students. The 24-item nursing decision-making scale is an abbreviated version of the original scale with 56 items. The participants might obtain a score within 24–120. A score under 67 shows analytic-systematic decision-making skills; a score within 68–78 indicates the second level of decision-making skills as analytic-intuitive; a score above 78 demonstrates the third level of decision-making skills as intuitive-interpretive. Analytic and intuitive are two extremes of the continuum of decision-making. Analytic-intuitive decision-making includes the ability to connect previous learnings to current perceptions regarding a clinical scenario and relies on the perception and comprehension of the information collected from several current and past sources of information. However, the analytic-systematic process is a linear method for decision-making concerning a problem. On the other hand, intuitive-interpretive decision-making is a combination of the aforementioned two [26]. This instrument is available in Persian and has been utilized in various researches done in Iran. Using the perspectives of Educational Psychology and Medical Education Professors in three categories, including relevance, clarity, and simplicity, Noohi et al. proved the content validity of this scale in Iran. They used both external reliability and the internal reliability of subscales to establish the reliability of the tool. The test-retest correlation was 0.90, and the Kappa coefficient was 0.83. As a result, the scale had acceptable validity and reliability [27]. Another research employed the test-retest approach to assess the reliability of this instrument, and the result was a Cronbach’s alpha coefficient of 0.86 [28].

The third section consisted of the self-efficacy in clinical performance scale (SECP) designed in Iran by Cheraghi et al. with 37 items based on the nursing context in four domains, namely “assessment” (12 items), “diagnosis and planning” (9 items), “implementation” (10 items), and “evaluation” (6 items), based on a 5-point Likert scale ranging from one (no confidence) to five (complete confidence). The total score is within the range of 37–185. The content validity and the face validity of the scale were examined by twenty nursing specialists from nursing faculties. The dimensions’ Cronbach’s alpha varied from 0.90 to 0.92, while the entire scale’s internal reliability was α = 0.96. A 2-week gap between tests resulted in a test-retest reliability of r = 0.94. Concurrent validity was also obtained and r and p were 0.73, and 0.01, respectively [29]. In the current study, Cronbach’s alpha coefficient of the scale was calculated as 0.89, indicating that it was reliable.

### Data analysis

The collected data were analyzed using descriptive statistics, the Mann-Whiney U test, paired and independent t-tests, and the Wilcoxon test. The differences in dependent variables in the initial stage and after the intervention were analyzed using a paired t-test, and the differences between groups were measured using the independent t-test. The Wilcoxon test was used to measure CDM levels in the group before and after the intervention. For the assessment of the differences in the levels of CDM between groups following the intervention, the Mann-Whitney U test was utilized. The data were analyzed using SPSS software (version 21).

## Results

The mean age of the students in the control and intervention groups were 21.89 ± 1.11 and 21.51 ± 1.01 years, respectively. In both groups, 51.4% and 48.6% of the participants were in their fourth and third years, respectively. The majority of the participants were female (80%). Furthermore, In the control group, 45.7% were satisfied with the anesthesiology nursing major, and 62.9% were content with practical training courses. According the results of a poll on students, all of them (100%) considered training sessions for SECP and CDM as necessary. Amongst the study subjects in the intervention group, 48.6% were satisfied with the anesthesiology nursing major, and 57.1% were content with practical training courses. In the classification of the students’ academic performance based on the total scores, the scores of 17–20, 14–17, and under 14 were considered high, average, and weak, respectively. Accordingly, the findings of the present study showed that 48.6% and 45.7% of the students in the control and intervention groups had average academic performance. According to the Fisher’s exact and Chi-square tests, both the intervention and control groups were similar and homogenous in terms of demographic characteristics (Table 2).

**Table 2:** Participants’ demographics

Variables	Categories	Cont. (n = 35)	Int. (n = 35)	${\varvec{x}}^{2} \varvec{o}\varvec{r} \varvec{t}$			P
		N (%) or Mean ± SD					
Age (years)		21.89 ± 1.11	21.51 ± 1.01	1.467			0.147
Academic year	Fourth-year	18 (51.4)	18 (51.4)				0.594^a^
	Third-year	17 (48.6)	17 (48.6)				
Gender	Male	7 (20)	7 (20)				0.617^a^
	Female	28 (80)	28 (80)				
Academic Performance	High level	10 (28.6)	8 (22.9)	0.726			0.696
	Average Level	17 (48.6)	16 (45.7)				
	Weak Level	8 (22.9)	11 (31.4)				
Satisfaction with Anesthesiology Nursing Major	Satisfied	16 (45.7)	17 (48.6)	0.275			0.872
	Moderate	14 (40)	12 (34.3)				
	Dissatisfied	5 (14.3)	6 (17.1)				
Satisfaction with Practical Training Courses	Satisfied	22 (62.9)	20 (57.1)	0.317			0.853
	Moderate	8 (22.9)	10 (28.6)				
	Dissatisfied	5 (14.3)	5 (14.3)				
Necessity of CDM Education	Necessary	35 (100)	35 (100)				
	Not necessary	0 (0)	0 (0)				
Necessity of SECP Education	Necessary	35 (100)	35 (100)				
	Not necessary	0 (0)	0 (0)				
Experiences of CDM Education	Yes	7 (20)	5 (14.3)	0.402			0.526
	No	28 (80)	30 (85.7)				
Experiences of SECP Education	Yes	5 (14.3)	6 (17.1)	0.108			0.743
	No	30 (85.7)	29 (82.9)				

Int. = intervention group; Cont. = control group

^a^ Fisher’s exact test

Prior to the intervention, the mean CDM scores were 66.60 ± 5.42 and 66.54 ± 3.69 in the control and intervention groups, respectively. After the training course, the scores in the intervention group were increased to 75.31 ± 7.72 (P < 0.001). Moreover, in the intervention group, according to paired t-test results, there were significant differences before and after the intervention (P < 0.001) (Table 3). The CDM has an Effect Size Glass’s delta = 2.183 and a power = 99.99%.

**Table 3:** Comparison of CDM Within- and Inter- group before and after the intervention

Groups	Control	Intervention	The independent–sample t test	
**Score of CDM**	**Mean ± SD**		t	P
**Pre intervention**	66.60 ± 5.42	66.54 ± 3.69	0.052	0.959
**Post intervention**	65.51 ± 4.49	75.31 ± 7.72	6.494	< 0.001
**The paired sample t test**	t = 1.197, P = 0.240	t = 6.460, P < 0.001		

*Independent–sample t test, Paired sample t test

Prior to the intervention, the mean SECP scores of the students in the control and intervention groups were 83.94 ± 15.51 and 85.97 ± 14.18, respectively (P = 0.570). Following the intervention, the scores of the nursing students in the intervention group were increased to 140.66 ± 22.43 (P < 0.001). Moreover, the mean scores of students in the intervention group were increased in all dimensions of the SECP scale (P < 0.001). The highest and lowest increases in the students’ mean scores of self-efficacy subscales were observed in the first and fourth dimensions, respectively (Table 4). The SECP has an Effect Size Glass’s delta = 3.048 and a power = 100%.

**Table 4:** Comparisons of SECP and their Subscales Within- and Inter- group before and after the intervention

Characteristics	Groups	Pre-test	Post-test	t	P
		Mean ± SD			
1. **SECP**	Cont.	83.94 ± 15.51	85.34 ± 18.15	0.391	0.698
	Int.	85.97 ± 14.18	140.66 ± 22.43	12.326	< 0.001
		t = 0.571, P = 0.570	t = 11.340, P < 0.001		
**1.1 Assessment**	Cont.	25.97 ± 6.42	25.94 ± 6.78	-0.022	0.982
	Int.	26.86 ± 9.46	42.51 ± 7.38	9.630	< 0.001
		t = 0.458, P = 0.648	t = 9.786, P < 0.001		
**1.2 Diagnosis & Planning**	Cont.	20.00 ± 4.85	19.11 ± 6.02	-0.782	0.439
	Int.	21.49 ± 6.05	34.74 ± 7.02	9.416	< 0.001
		t = 1.134, P = 0.261	t = 9.997, P < 0.001		
**1.3 Implementation**	Cont.	24.37 ± 5.15	24.86 ± 5.61	0.422	0.676
	Int.	24.03 ± 4.02	37.91 ± 6.46	12.106	< 0.001
		t= -0.311, P = 0.757	t = 9.028, P < 0.001		
**1.4 Evaluation**	Cont.	12.49 ± 3.31	12.66 ± 3.64	0.298	0.768
	Int.	13.89 ± 3.98	23.14 ± 4.64	8.827	< 0.001
		t = 1.601, P = 0.114	t = 10.520, P < 0.001		

Int. = intervention group; Cont. = control group

*Independent–sample t test, Paired sample t test

The results of the Mann-Whitney U test demonstrated that CDM skills were significantly promoted, after the training program, in the intervention group in comparison to those of the control group (P < 0.001). Moreover, according to the results of the Wilcoxon test, there was a significant difference in the intervention group before and after the intervention (P < 0.001); accordingly, the CDM skills of the students were promoted to the next level (Table 5).

**Table 5:** Comparison of level of CDM between the two groups

Time	Level of CDM	Cont.	Int.	Mann-Whitney U test	
		N (%)		Z	P
**Pre intervention**	Analytic-systematic	24 (68.6)	21 (60.0)	-0.743	0.458
	Analytic-intuitive	11 (31.4)	14 (40.0)		
	Intuitive-interpretive	0 (0)	0 (0)		
**Post intervention**	Analytic-systematic	23 (65.7)	5 (14.3)	-4.708	< 0.001
	Analytic-intuitive	12 (34.3)	22 (62.9)		
	Intuitive-interpretive	0 (0)	8 (22.9)		
**Wilcoxon test**		Z= -0.333	Z= -4.179		
		P = 0.739	P < 0.001		

Int. = intervention group; Cont. = control group

*Mann-Whitney U test, Wilcoxon test

## Discussion

The current study was conducted with the main objective of the assessment of the effectiveness of the implementation of an SBAR-based course in SECP and CDM skills of anesthesiology nursing students.

The results of the current study indicated that the implementation of SBAR-based training courses could improve the average scores and promote the levels of CDM skills in anesthesiology nursing students. One of the most important findings of the current study was that the frequency distribution of the intuitive-interpretive decision-making skills was elevated from 0 to 22.9% in the intervention group. In the control group, 68.6% and 31.4% of the students had analytic-systematic and analytic-intuitive decision-making skills, respectively. These levels were only slightly improved following the intervention. In the intervention group, the distribution of analytic-systematic skills decreased from 60 to 14.3%. However, the distribution of the levels of analytic-intuitive and intuitive-interpretive skills were promoted from 40% and 0 to 62.9% and 22.9%, respectively.

None of the previous studies has assessed the efficiency of the implementation of SBAR-based training courses on SECP and CDM skills of anesthesiology nursing students. Nonetheless, Cho et al. (2020) demonstrated that simulation training based on SBAR could promote communication skills and clinical decision-making skills of nursing students [30]. Oh et al. (2021) showed that SBAR-based simulation learning could promote the clinical judgment of nursing students and improve confidence in inter-professional communication [31]. The aforementioned findings are in line with the results of the present study.

Among other findings of the current study, this might be highlighted that SBAR-based training could promote SECP scores in the anesthesiology nursing students. In a study conducted by Do et al. (2019), it was demonstrated that SBAR-based training is effective in the promotion of self-efficacy of nursing students [32]. The results of another study by Kim et al. (2016) showed that SBAR could improve nurses’ clinical competence and self-efficacy. Moreover, they recommended that organizations should obtain the benefits of this program in inter-professional relationships, clinical competence, and self-efficacy in nursing tasks [19], which is in line with the findings of the current study.

As a strategy for the rapid organization of patient information, SBAR facilitates the interpretation of results. Moreover, SBAR can promote clinical judgment capability through the process of reflecting clues or anticipated results in patients’ conditions [33]. Yoon et al. (2018) recommended using the SBAR method as a “fundamental nursing education method” for nursing students. They also declared that SBAR could be considered a teaching method for nursing students [34]. In the current study, it appears that understanding patients’ conditions, priority-based analysis of health issues, and planning and executing nursing activities can be effective in a simulation setting similar to the clinical environment of the operation theater. Moreover, curriculum strategies to improve readiness include extra clinical hours and simulation experiences [35]. Therefore, as the implementation of SBAR can promote SECP and CDM skills amongst students, it should be added to the curriculum of undergraduate nursing students as an effective teaching strategy.

The results of a study carried out by Hsu et al. (2015) demonstrated that scenario-based simulation techniques could promote satisfaction, improve communication skills, and be used as a training method for the promotion of communication competence of in-service nurses [36]. In a meta-analysis, Shin et al. (2015) showed that simulation strategies are more effective in the promotion of learning than traditional methods [37]. The present study utilized simulation and role-playing methods for the promotion of SECP and CDM skills in anesthesiology nursing students. Hence, considering the increase in SECP and CDM skills, it is suggested to use an SBAR training course in future studies to demonstrate its possible effectiveness in the improvement of the quality of clinical training and elevation of clinical competence.

### Study limitations

One of the main limitations of the current study was its small sample size. Performing the study at only one university can also impede generalizability. Another limitation could be the use of only one assessor for the evaluation of participants.

## Conclusion

The application of SBAR during a clinical training course can be considered an important and effective educational strategy for the complete achievement of educational objectives in a simulated clinical setting. The present study used an SBAR education technique to develop clinical decision-making and self-efficacy. The results showed that the CDM and SECP scores of participants in the intervention group were significantly higher than those in the control group. It is suggested that all surgical team professionals participate in SBAR education.

## Data Availability

The datasets used and/or analyzed during the current study are available from the corresponding author on reasonable request.

## Abbreviations

SBAR: Situation, Background, Assessment, and Recommendation
CDM: Clinical Decision-Making
SECP: Self-Efficacy in Clinical Performance

## References

[1] Shahijani G, Ghadami A, Shakerian M (2019). A comparative study on the risk factors of Musculoskeletal Disorders among Laparoscopic Surgical Technologists in Circulatory and Scrub Roles. BRAIN Broad Research in Artificial Intelligence and Neuroscience.

[2] Heshmati H (2015). Effective factors in clinical education quality from the viewpoints of operation room and anesthesiology students in Torbat Heydarieh University of Medical Sciences. Iran J Med Educ.

[3] Etemadifar S, Sedighi Z, Masoudi R, Sedehi M (2020). Evaluation of the effect of SBAR-based patient safety training program on nurses’ clinical decision-making in the intensive care unit. J Clin Nurs Midwifery.

[4] Moghadam S, Manzari SZ, Motlagh G (2017). Clinical decision making of nurses in intensive care units in Mashhad teaching hospitals. J Sabzevar Univ Med Sci.

[5] Kouravand Z, Aein F, Ebadi A, Yadegarfar G. Nurses’ perception of clinical decision making in hospitals of Shahrekord University of Medical Sciences in 2019.Journal of Clinical Nursing and Midwifery2019, 8(3).

[6] Al-Hamdan ZM, Bawadi HA, Redman RW, Al-Nawafleh AH (2016). Perception of jordanian nurses regarding involvement in decision-making. Appl Nurs Res.

[7] Alizadeh I, Salari A, Ahmadnia Z, Moaddab F. An Investigation into Self-efficacy, Clinical Decision-making and the Level of Relationship between them among Nurses in Guilan Province. 2020.

[8] Sung S-C, Huang H-C, Lin M-H (2015). Relationship between the knowledge, attitude, and self-efficacy on sexual health care for nursing students. J Prof Nurs.

[9] Labrague LJ, McEnroe-Petitte DM, Bowling AM, Nwafor CE, Tsaras K. High‐fidelity simulation and nursing students’ anxiety and self‐confidence: A systematic review. Nursing Forum: 2019:Wiley Online Library; 2019:pp. 358–368. 10.1111/nuf.12337 30852844

[10] Baaij A, Özok A, Vaeth M, Musaeus P, Kirkevang LL (2020). Self-efficacy of undergraduate dental students in endodontics within Aarhus and Amsterdam. Int Endod J.

[11] Lee J (2021). Situation, background, Assessment, and recommendation Stepwise Education Program: a quasi-experimental study. Nurse Educ Today.

[12] Chae M (2019). The effect of simulation-based SBAR education programs of nursing students. Indian J Public Health Res Dev.

[13] Jeong JH, Kim EJ (2020). Development and evaluation of an SBAR-based fall simulation program for nursing students. Asian Nurs Res.

[14] Kim S, Kim H (2020). Effects of the SBAR training program in essential fundamental nursing skills on communication competence, critical thinking disposition and problem-solving ability of nursing students. J Learner-Centered Curriculum Instruction.

[15] Yu M, ja Kang K (2017). Effectiveness of a role-play simulation program involving the sbar technique: a quasi-experimental study. Nurse Educ Today.

[16] Lee H, Jang SJ (2021). Effects of flipped-learning‐based simulation for nursing students: a retrospective survey. Int J Ment Health Nurs.

[17] Raksamani K, Jirativanont T, Sareenun P (2020). Correlation of medical knowledge and non-technical skills assessment in anesthesia residents. Siriraj Med J.

[18] Kesten KS (2011). Role-play using SBAR technique to improve observed communication skills in senior nursing students. J Nurs Educ.

[19] Kim YH, Choi YS, Jun HY, Kim MJ (2016). Effects of SBAR program on communication clarity, clinical competence and self-efficacy for nurses in cancer hospitals. Korean J Rehabilitation Nurs.

[20] Yuliyanti R, Arso SP, Ardani MH (2020). Increasing job satisfaction of nurses through SBAR communication in handover of nursing tasks. Jurnal Aisyah: Jurnal Ilmu Kesehatan.

[21] Kostoff M, Burkhardt C, Winter A, Shrader S. An interprofessional simulation using the SBAR communication tool.American Journal of Pharmaceutical Education2016, 80(9). 10.5688/ajpe809157 PMC5221839 28090106

[22] Gamboa OA, Agudelo SI, Maldonado MJ, Leguizamón DC, Cala SM (2018). Evaluation of two strategies for debriefing simulation in the development of skills for neonatal resuscitation: a randomized clinical trial. BMC Res Notes.

[23] Wunder L, Gomez NAG, Gonzalez JE, Mitzova-Vladinov G, Cacchione M, Mato J, et al. editors. Fire in the operating room: use of mixed reality simulation with nurse anesthesia students. *Informatics* 2020, MDPI.

[24] Huang P, Jensen S. Application of the science of learning to nurse anesthesia students to improve self-efficacy: a pilot study. Rutgers University-School of Nursing-RBHS; 2019.

[25] Lauri S, Salanterä S (2002). Developing an instrument to measure and describe clinical decision making in different nursing fields. J Prof Nurs.

[26] Phillips BC. Clinical decision making in last semester senior baccalaureate nursing students. The University of Wisconsin-Milwaukee; 2015.

[27] Noohi E, Karimi-Noghondar M, Haghdoost A (2012). Survey of critical thinking and clinical decision making in nursing student of Kerman University. Iran J Nurs midwifery Res.

[28] Ahmady S, Hosseini TM. Effectiveness of Video-assisted Debriefing Versus Standard Oral Debriefing Following Screen-based Simulation (CyberPatient TM) Training.Journal of Medical Education2022, 21(1).

[29] Cheraghi F, Hassani P, Yaghmaei F, Alavi-Majed H (2009). Developing a valid and reliable self‐efficacy in clinical performance scale. Int Nurs Rev.

[30] Cho H-H, Nam K-h, Park J-S, Jeong H-E, Jung Y-J (2020). The effect of simulation training applying SBAR for nursing students on communication clarity, self-confidence in communication, and clinical decision-making ability. J Korea Academia-Industrial cooperation Soc.

[31] Oh H (2021). Effects of Simulation Learning using SBAR on Clinical Judgment and Communication Skills in undergraduate nursing students. Int J Contents.

[32] Do J, Shin S (2019). Development of handoff education program using SBAR for nursing students and its effect on self-efficacy, communication ability and clinical performance ability. J Korean Acad Fundamentals Nurs.

[33] Lancaster RJ, Westphal J, Jambunathan J (2015). Using SBAR to promote clinical judgment in undergraduate nursing students. J Nurs Educ.

[34] Yoon J-H, Lee E-J (2018). The effect of team based simulation learning using SBAR on critical thinking and communication clarity of nursing students. J Korea Academia-Industrial cooperation Soc.

[35] Woods C, West C, Mills J, Park T, Southern J, Usher K (2015). Undergraduate student nurses’ self-reported preparedness for practice. Collegian.

[36] Hsu L-L, Chang W-H, Hsieh S-I (2015). The effects of scenario-based simulation course training on nurses’ communication competence and self-efficacy: a randomized controlled trial. J Prof Nurs.

[37] Shin S, Park J-H, Kim J-H (2015). Effectiveness of patient simulation in nursing education: meta-analysis. Nurse Educ Today.

